# A Survey on Tubulin and Arginine Methyltransferase Families Sheds Light on *P. lividus* Embryo as Model System for Antiproliferative Drug Development

**DOI:** 10.3390/ijms20092136

**Published:** 2019-04-30

**Authors:** Maria Antonietta Ragusa, Aldo Nicosia, Salvatore Costa, Caterina Casano, Fabrizio Gianguzza

**Affiliations:** 1 Department of Biological, Chemical and Pharmaceutical Sciences and Technologies (STEBICEF), University of Palermo, Viale delle Scienze, Ed. 16, Palermo, 90128 Sicily, Italy.; salvatore.costa@unipa.it (S.C.); casanocaterina@gmail.com (C.C.); fabrizio.gianguzza@unipa.it (F.G.); 2 National Research Council-Istituto per lo studio degli impatti Antropici e Sostenibilità in ambiente marino (IAS-CNR), Laboratory of Molecular Ecology and Biotechnology, Detached Unit of Capo Granitola, Via del mare, Torretta Granitola (TP), 91021 Sicily, Italy; aldo.nicosia@cnr.it

**Keywords:** tubulin, PRMT, post-translational modification, arginine methylation, sea urchin, echinoderms

## Abstract

Tubulins and microtubules (MTs) represent targets for taxane-based chemotherapy. To date, several lines of evidence suggest that effectiveness of compounds binding tubulin often relies on different post-translational modifications on tubulins. Among them, methylation was recently associated to drug resistance mechanisms impairing taxanes binding. The sea urchin is recognized as a research model in several fields including fertilization, embryo development and toxicology. To date, some α- and β-tubulin genes have been identified in *P. lividus*, while no data are available in echinoderms for arginine methyl transferases (PRMT). To evaluate the exploiting of the sea urchin embryo in the field of antiproliferative drug development, we carried out a survey of the expressed α- and β-tubulin gene sets, together with a comprehensive analysis of the PRMT gene family and of the methylable arginine residues in *P. lividus* tubulins. Because of their specificities, the sea urchin embryo may represent an interesting tool for dissecting mechanisms of tubulin targeting drug action. Therefore, results herein reported provide evidences supporting the *P. lividus* embryo as animal system for testing antiproliferative drugs.

## 1. Introduction

It is well known that different α- and β-tubulin isotypes contribute to create an evolutionary conserved network of highly dynamic filaments, with key roles in cellular architecture and physiology [1].

The α- and β-tubulin dimers linearly assemble to create polarized proto-filaments with β-tubulin subunits exposed to the solvent at the plus end, while the minus-end is capped by α-tubulin subunits [2,3]. Classically, 13 proto-filaments are known to assemble into an MT, a cylinder of 22 nm in diameter. MTs are responsible for several aspects of cell division and proliferation as well as development and tissue differentiation, signalling pathways and gene expression modulation [4,5,6]. Beyond the role in organizing chromosome movements during cell divisions [7], MTs interacting with kinetochore are responsible for the mechanism of genome surveillance known as spindle assembly checkpoint [8]. Changes in kinetochore MT stability often result in chromosome segregation errors, aneuploidy and tumorigenesis [9,10].

Alteration of the dynamic instability between free and polymerized tubulin, as a result of antitumor drugs that interact with MTs and tubulin, severely affects the cell fate leading apoptosis after cell cycle arrest at G2/M [11,12], thus depicting MTs as drug targets in cancer treatment.

Interestingly, several lines of evidence unveiled a role for the switch of tubulin isotypes [13] directly affecting mechanisms of tumorigenesis and resistance to tubulin-binding chemotherapy agents [14,15,16]. Moreover, tubulins can be post-translationally chemically modified changing their affinity towards cellular interactors and their binding to different drugs [17,18,19,20]. All that could lead to a regulation of cell activities and cell cycle control, and a different response to chemotherapeutic agents [10,21,22,23].

The α- and β-tubulins constitute a multigene family in several species. At least 4 α- and 4 β-tubulin genes were found in *Drosophila melanogaster*, in different members of hemimetabolous and holometabolous [24]; while the human genome contains at least 15 α-tubulin genes and 21 β-tubulin genes which are on distinct chromosomes and possess specific tissue and developmental distributions [25,26]. Among Echinodermata, genome wide analyses of the sea urchin *Strongylocentrotus purpuratus* retrieved 9 α-tubulin genes and 6 β-tubulin genes [27], thus providing evidences for the existence of multiple gene families also in non-chordates deuterostomes.

Sea urchin is recognized as a research model for fertilization [28], mechanisms of embryo development and specification [29,30,31], bilaterian development [32], gene regulatory networks [33,34] and stress responses [35,36]. Interestingly, the first hypothesis on tumorigenesis was provided by Boveri analysing alteration in the mitotic apparatus during sea urchin cleavage [37,38]. Moreover, the sea urchin embryo was also the perfect model for cyclin discovery [39,40].

Because of the synchrony in cleavage times related to the correlation between cell cycle regulation and mitotic apparatus of the sea urchin embryo system, tubulins and tubulin targeting drugs may represent an interesting tool for analyse antimitotic molecules that affect tubulin dynamics and drug activity on MT assembly and stability [41]. In fact, during development two distinct processes are directly connected to microtubule dynamics: the early cleavage and the ciliary dependent swimming which occurs later in development. Among drugs interacting with MTs, taxanes are widely studied both as tool for experimental researches and as antiproliferative and chemotherapeutic agents. The effects of taxol were observed on the sea urchin embryo mitotic apparatus and in particular the alterations of cleavage furrow during blastomere segmentation were reported [42]. Moreover, a sea urchin embryo-based protocol for the assessment of multiple tubulin destabilizing drugs has been already proposed [41] and successfully used in several studies [43,44,45].

To date, some α- and β-tubulin genes have been identified and characterized from the Mediterranean sea urchin *Paracentrotus lividus* [46,47,48,49,50,51]; and mechanisms of transcriptional regulation have been finely defined for the neural α-tubulin [52,53,54]. However, to date a comprehensive view of tubulins and MTs related to post-translational modifications (PTMs) and especially arginine methylation is still lacking.

During the last few years, several *P. lividus* transcriptome datasets have been generated [55,56], thus allowing the identification of other gene families [57,58]. While, regarding arginine methyl transferases (PRMTs), no data are still available in echinoderms. Therefore, in the present work, we carried out a survey of the expressed α- and β-tubulin gene sets, together with a comprehensive analysis of the PRMT gene family and the predicted methylable arginine residues in *P. lividus* tubulins. This will provide the basal elements for a tool kit to study arginine methylation sensitive drugs.

## 2. Results and Discussion

### 2.1. P. lividus α- and β-Tubulin Identification and Their Predicted PTMs

The availability of large-scale transcriptome collections freely available on public databases allowed us to carry out a transcriptome survey in the sea urchin embryo *P. lividus.* To identify the expressed α- and β-tubulin multigene family, we implemented BLAST searches. Given the high similarity of α- and β-tubulin sequences, each identification was manually curated as well as reconfirmed by comparative analysis.

Starting from collected sequences in the EST databases, specific primer sets were designed and used to isolate the 3′- and 5′-ends of the cDNAs. The full-length cDNAs were obtained by assembling the 3′ and 5′ RACE products with the original sequences and were validated by sequencing. Several other predicted homologues were detected in the database but were not subjected to further analysis as they contained truncations or domain insertions. To avoid confusion in nomenclature, we used the tubulin gene names coined in the purple sea urchin *S. purpuratus* or in previous reports [27]. Moreover, sequences corresponding to Tuba1a (α2), Tuba1g (α10), Tuba1h (α1), Tubb2a (β3), Btub2 (β2) and Btub5 (β1) derive from already isolated tubulin transcripts or genes [46,47,48,49,50,51,52,53,54].

All the transcripts contain the Kozak consensus surrounding the initiator codon, while stop codons, polyadenylation signals and a poly(A) tail were found in the 3′-UTRs (untranslated region). The length of mRNAs, open reading frames (ORFs), corresponding amino acid residues and theoretical parameters for each of them are summarized in Table 1.

**Figure 1:**
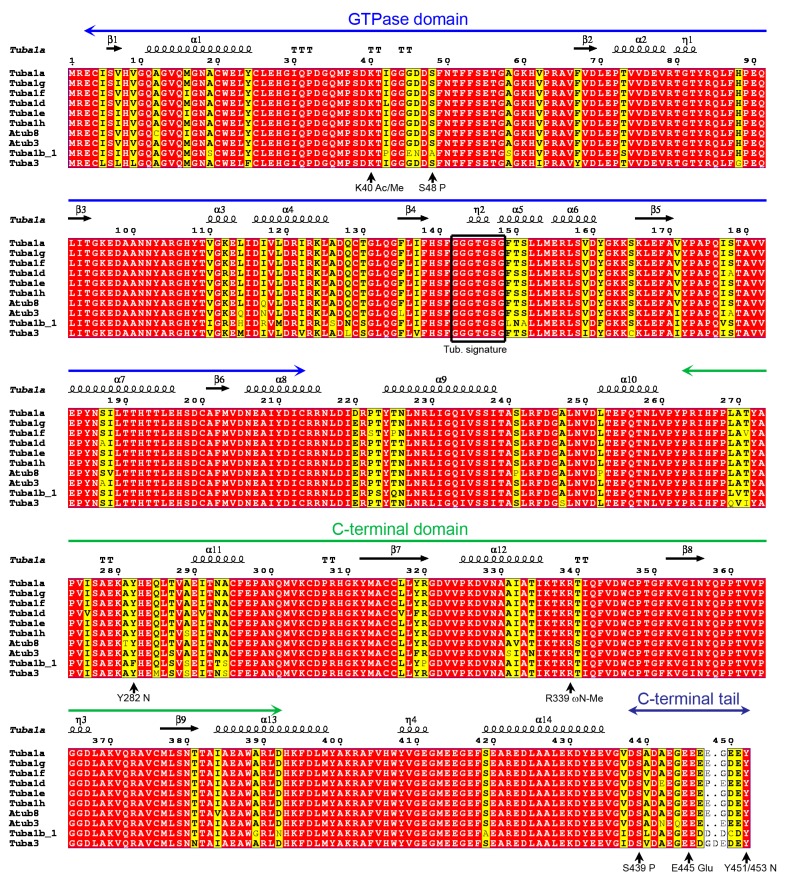
Multiple sequence alignment (MSA) of the α-tubulins of *P. lividus*. Alignment was performed with T-coffee and rendered by ESPript 3.0. Similar residues are written in black bold characters and boxed in yellow whereas conserved residues are in white bold characters and boxed in red. The sequence numbering on the top refers to the alignment. Tubulin domains are highlighted by long arrows also on the top. The principals PTMs are indicated on the bottom. Ac: acetylation; Me: methylation; P: phosphorylation; Glu: polyglutamylation and polyglycylation.

**Figure 2:**
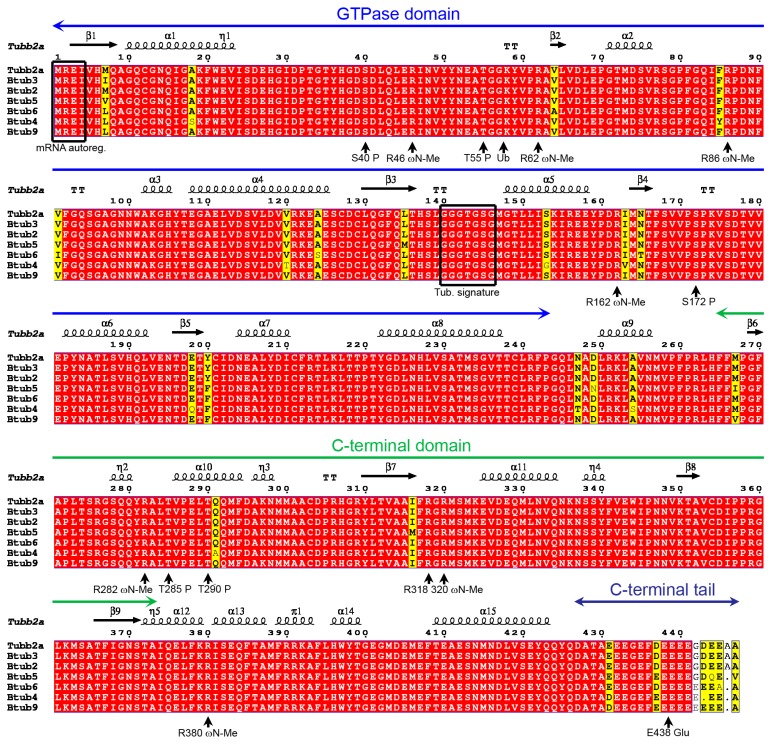
MSA of the β-tubulins of *P. lividus*. Alignment was performed with T-coffee and rendered by ESPript 3.0. Similar residues are written in black bold characters and boxed in yellow whereas conserved residues are in white bold characters and boxed in red. The sequence numbering on the top refers to the alignment. Tubulin domains are highlighted by long arrows also on the top. The principals PTMs are indicated on the bottom. Me: methylation; P: phosphorylation; Glu: polyglutamylation and polyglycylation.

The *P. lividus* α- and β-tubulins are organized in an N-terminal GTPase domain with the canonical tubulin signatures GGGTGSG mapping the amino acid residues 142–148 in α- and 140–146 in β-tubulins, respectively, and a C-terminal domain connected by a central helix (Figure 1 and Figure 2). Moreover, tubulins possess an acidic and flexible Carboxy-terminal tail of about 10–15 amino acid residues that in the MTs decorates the external surface and through which MTs interact with microtubule-associated proteins (MAPs) [59].

A computational search for PTMs identified several conserved Ser/Thr phosphorylation recognition sites including S48 and S439 in the α-tubulin chains as well S40, S172, T55, T285 and T290 in β-tubulins (Figure 1 and Figure 2). Even if the functions of these PTMs have remained almost elusive for years, new lines of evidence suggested a control of the dimerization dynamic and a role in the MT behaviour during cell division, especially for β-tubulin phosphorylation at S172 by cyclin-dependent kinase 1 [19,60].

Acetylation of α-tubulin on lysine 40 represents a common PTM especially found on stable MTs [61,62], making microtubules more resistant to mechanical forces [63,64]. Our analyses confirmed the presence of a specific amino acid residue (K40) acetylation/deacetylation site in all α-tubulin (Figure 1) but not β-tubulin isoforms. Similarly, only the α-tubulins contain a Tyr residue (Y451/453) at the C-terminus which may undergo detyrosination/tyrosination cycles corresponding to assembled or disassembled MTs [20,62]. Interestingly, detyrosination guides chromosome congression during mitosis [65]. Together with K40 acetylation, detyrosination was shown to regulate Kinesin-dependent transport [66].

Additionally, exclusively the β-tubulin isoforms possess the N-terminal tetrapeptide (MREY) which co-translationally regulates the mRNA degradation by negative β-tubulin autoregulatory binding (Figure 2), thus altering mRNA stability [67].

In the tubulin dimer, the C-terminal tail harboured by α- and β-tubulins is exposed to the solvent allowing polyglutamylation [68,69] and it is involved in the regulation of interactions between MTs and different structural and motor MAPs, the regulation of flagellar and ciliary beating and the stability of centrioles/centrosomes. Similarly, polyglycylation also occurs at the C-terminal tail and it has been implicated in the mechanical stabilization of the axoneme [20,70].

Polyglutamylation has been studied also in *P. lividus*, establishing that this PTM is virtually absent in β-axonemal tubulins and that α-tubulin polyglutamylation (that is not evenly distributed along the flagellar length) is important for flagellar motility [71,72,73].

Computational analyses on sea urchin tubulins mapped the existence of polyglutamylation sites in all the proteins, including E445 and E438 in α- and β-tubulins respectively (Figure 1 and Figure 2). This confirms the regulatory role ascribed to these PTMs, connecting tubulins to their associated proteins [20].

Several other PTMs have been identified on tubulins, including ubiquitination and methylation [74]. However, few details on related functions have been provided to date. Interestingly, on the basis of the computational analysis, β-tubulins contain the glycyl-lysine isopeptide (G57K58) that putatively undergoes ubiquitination (Figure 1 and Figure 2).

Recently, methylation has been found on tubulins occurring in lysine and arginine amino acid residues [22,75,76,77,78,79,80] and it has not been characterized in detail.

All these features, including post-transcriptional and post-translation mechanisms, represent a hallmark of the tubulin multigene family and the polymer structural diversity [74,75,76].

A MSA analysis confirm the high similarity among members of α- and β-tubulin family and that the main differences located at the C-terminal tail may correspond to the differential isotype class involved in specific heterodimers which in turn may affect MT properties. Additionally, it appears that α isotypes accept more residue variations in the corresponding sequence. An analysis of amino acid substitutions shows that, both in α and β isotypes, about 50% of variability occurs in α helix or β sheet secondary structures. The additional variations showed by α isotypes mainly involve amino acid residues structured in α helix. This is not surprisingly since α helices were already found underrepresented in conserved regions [81,82].

### 2.2. P. lividus α- and β-Tubulin Gene Organization

To characterize the α- and β-tubulin gene structures, efforts were made to isolate genomic loci related to cDNAs.

**Figure 3:**
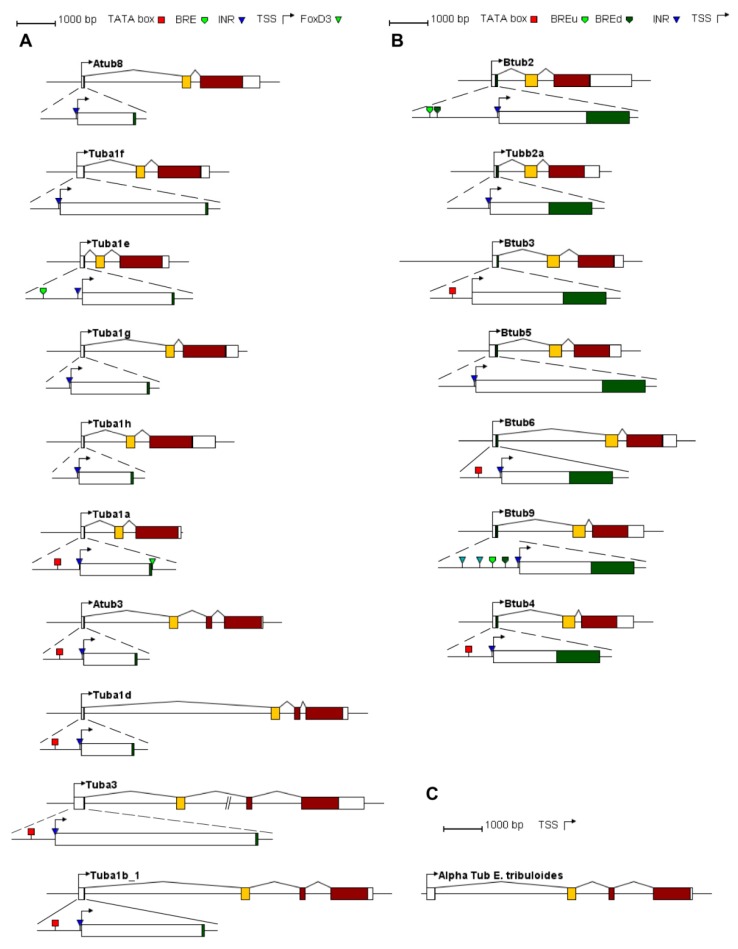
α- and β-tubulin gene structure and core promoter analysis. Schematic gene structures of the *Paracentrotus lividus* α-tubulins (**A**), β-tubulins (**B**) and an α-tubulin gene of *Eucidaris tribuloides* (**C**), drawn to scale. The bent arrows indicate the putative transcription start sites (TSS). Boxes represent exons. In particular, white boxes indicate untranslated regions and coding regions are coloured. Core promoter elements are shown as indicated.

Seven β-tubulin genomic clones and 11 diverse clones corresponding to α-tubulins were selected. The gene structures of the *P. lividus* tubulin genes are represented in Figure 3A,B.

The comparison between cDNA and genomic sequences revealed that the transcription unit of all the β-tubulin genes and α genes including Atub8, Tuba1f, Tuba1e, Tuba1g, Tuba1h and Tuba1a, are composed of three exons interrupted by two introns, whereas in Atub3, Tuba1d, Tuba3 and Tuba1b_1 an additional intron interrupts the coding sequence at the amino acid 125 (phase-0); thus the fourth exon includes sequences encoding the Tub signature, the remaining part of the GTPase domain and the C-terminal domain. In α-tubulin genes, the first intron occurs after the first codon (phase-0) and the second intron is located after the codon number 75 (phase-1). To verify if the third intron occurring in position 125 was acquired before the origin of Echinodermata phylum, an inspection of the α-tubulin expressed genes in a member of the basal echinoid order Cidaroida was performed. Indeed, an α-tubulin gene structured with four exons was found in the slate pencil urchin *Eucidaris tribuloides* and is reported in Figure 3C. Therefore, it could be reasonably hypothesized that this intron originated before the speciation of Echinodermata and was maintained in the lineage leading to Vertebrata [83].

In β-tubulin genes, the first intron occurs after the codon number 19 (phase-0) and the second intron is located after the codon number 131 (phase-0). All of them possess canonical splicing sites, identified at 5′-end by GT and at 3′-end by AG consensus sequences. Interestingly, the first intron is vertebrates specific, while the second one is shared with invertebrates and is maintained with the alga and fungi [83].

Moreover, computational analyses were performed on the proximal upstream region, revealing the presence of canonical sharp core promoter elements (BRE, TATA box and/or INR) often organized in couples, as reported in Figure 3 and Table 2.

**Figure 4:**
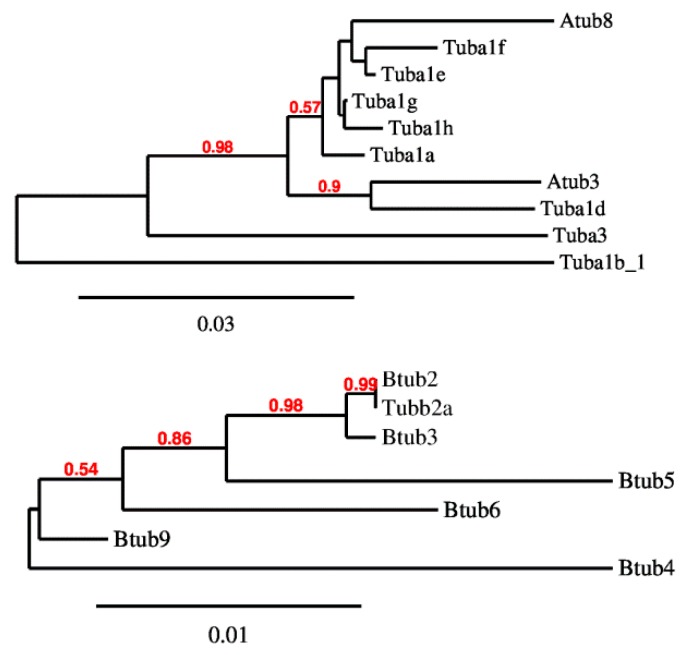
*P. lividus* α- and β-tubulin NJ distance trees. The trees were generated using MEGA X. The bootstrap consensus tree inferred from 1000 replicates is taken to represent the reliability of the branches of the analysed sequences. The percentage of replicate trees in which the associated taxa clustered together in the bootstrap test (1000 replicates) greater than 50% are shown in red next to the branches.

Both sequence comparison and gene structure analyses provide similar suggestions about the evolution of these gene families, in particular the isotypes that are closely related each other based on tree topology (Figure 4), show the same gene organization and promoter features. This is particularly evident for α-tubulin genes, indeed the divergent ones possess the additional intron and are TATA box dependent (Atub3, Tuba1d, Tuba3 and Tuba1b_1). Additionally, for β-tubulin genes is it possible to observe a stricter relation among genes showing similar first intron length (Btub2, Tubb2a and Btub3).

### 2.3. P. lividus α- and β-Tubulin Gene Expression

To analyse the expression profiles of α- and β-tubulin embryonic transcripts, total RNA was isolated from different developmental stages including eggs, early blastula, late blastula and prism and RT-qPCR were performed. The α- and β-tubulin gene families contain members whose transcripts are detected both in unfertilized eggs and during development, and genes whose transcripts showed different expression levels during embryogenesis.

Atub8, Tuba1g, Tuba1h and Tuba1b_1 transcripts resulted maternally inherited since they were detected in unfertilized eggs. Atub8, Tuba1g, Tuba1h resulted expressed also during the development reaching the higher levels at early/late blastula stages and then decreasing at prism, whereas Tuba1b_1 was not expressed during early development and was re-expressed at prism.

The expression of the remaining α-tubulin genes was developmentally regulated rising progressively until early and late blastula as occurred for Tuba1a and Tuba3 or restricted at late blastula stage (Tuba1f) or prism stage (Tuba1e, Atub3 and Tuba1d).

Among β-tubulin genes, only the Btub9 transcript was found in unfertilized eggs and its levels are high; while the mRNA level was reduced during the development. Btub2 and Btub3 transcripts showed similar profiles being expressed in a stage specific manner at early blastula and prism; while Btub6 and Btub4 mRNA expression was restricted at prism. Conversely, Tubb2a and Btub5 showed constitutive expression during development. The results agree with previous reports [47,48,49,50] and are summarized in Table 1.

### 2.4. P. lividus PRMT Orthologues

Due to the growing interest in the protein methylation effects and the recent discover of tubulin methylated sites in neural cells [22], together with the lack of information in the sea urchin methyl transferase, we studied the protein arginine methyltransferase family in *P. lividus*.

Using human PRMT protein sequences as queries, we detected in the *P. lividus* EST database partial sequences coding for PRMT1 to PRMT7. To complete coding sequences, 3′ RACE PCRs were performed. Clones selection was carried out in the same manner as for tubulin cDNAs. Predicted amino acid sequences, whom features are summarized in Table 3, were in silico characterized.

An effort was also carried out to isolate cDNAs coding for PRMT8 and PRMT9, however no matching sequences were retrieved in the dataset. Similarly, a screening of the cDNA and genomic libraries, using degenerate primer sets failed to detect any positive signal (data not shown). Therefore, we hypothesize that in *P. lividus* no orthologues of such methyltransferases do exist. The PMRT gene family and their related protein features are summarized in Table 3.

**Figure 5:**
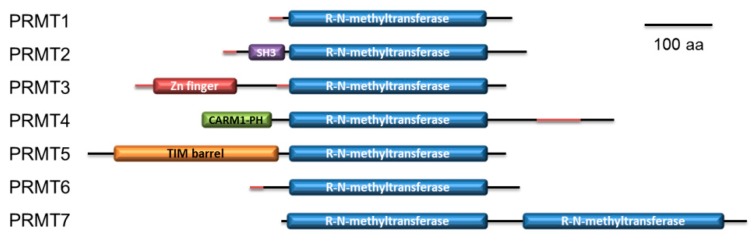
Schematic representation of the seven *P. lividus* PRMT isoforms and their domains predicted by InterPro (drawn to scale). SH3: SRC homology 3 domain; Zn finger: Zinc finger C2H2 superfamily; CARM1/PH: Histone-arginine methyltransferase CARM1, N-terminal / PH-like domain superfamily; TIM barrel: PRMT5, TIM barrel domain; R-N-methyltransferase: SAM-dependent methyltransferase PRMT-type domain. The protein regions predicted as disordered are depicted in red.

**Figure 6:**
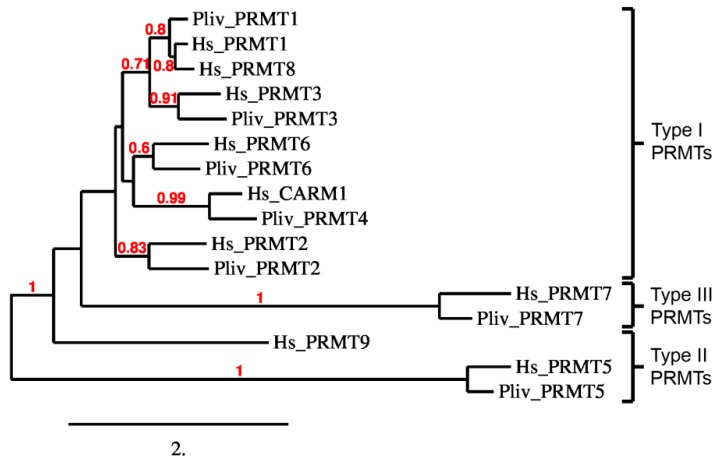
Amino acid sequences of *P. lividus* and *Homo sapiens* PRMTs were aligned and a distance tree was constructed. The tree was generated using MEGA X. The bootstrap consensus tree inferred from 1000 replicates is taken to represent the reliability of the branches of the analysed sequences. The percentage of replicate trees in which the associated taxa clustered together in the bootstrap test (1000 replicates) greater than 50% are shown in red next to the branches. Pairwise amino acid sequence alignment of human and *P. lividus* PRMTs are shown in Supplementary Materials Figures S1 and S2.

Inspection of the predicted domains of *P. lividus* PRMTs showed that they possess the canonical methyltransferase domain. Overall, a SAM-dependent methyltransferase PRMT-type domain is shared among them. In addition, a Src homology 3 (SH3) domain in PRMT2, a Zinc finger C2H2 superfamily domain in PRMT3, a Pleckstrin homology (PH) like domain similar to CARM1 N-terminal histone-arginine methyltransferase domain in PRMT4, a TIM barrel domain in PRMT5 and a second methyltransferase domain in PRMT7 were found (Figure 5). On the basis of domain organization, protein comparison with human orthologues (see supplemental material) and phylogenetic analysis (Figure 6), we likely hypothesize that sea urchin PRMT1, PRMT2, PRMT3, PRMT4 and PRMT6 belong to the type-I enzyme while PRMT5 to type-II and PRMT7 to type III [84].

**Figure 7:**
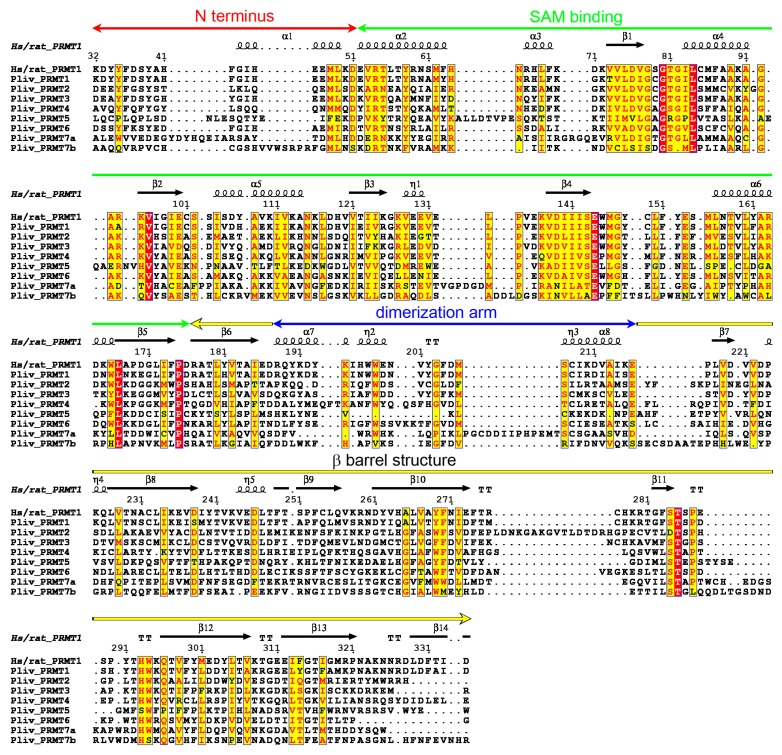
MSA of the methyltransferase domains (ProSite ID PS51678) of human and rat PRMT1 and *P. lividus* PRMT family performed with T-Coffee. Similar residues are written in black bold characters and boxed in yellow whereas conserved residues are in white bold characters and boxed in red. The sequence numbering on the top refers to the rat PRMT1 sequence (Q63009). PRMT1 domains and secondary structures are highlighted on the top; the colour coding is red for the N terminus (residues 41–51), green for the SAM binding domain (residues 52–176), yellow for the β barrel structure (residues 177–187 and 217–352), and blue for the dimerization arm (residues 188–216), as depicted in Zhang and Cheng [85].

An MSA of the arginine methyltransferase domains show the conservation between mammal and echinoderm PRMTs (see also Supplementary Figures S1 and S2) and the typical differences into the PRMT family. The active site residues, essential for enzymatic activity, are conserved (i.e., glutamates 144 and 153 in rat, 149 and 158 in *P. lividus*, Figure 7) as well as the GxGxG motif used to bind the adenosyl part of S-adenosyl-L-methionine (SAM) and the acidic residue at the end of β2 that forms hydrogen bonds to both hydroxyls of the SAM ribose (glutamate 100 in rat, 105 in *P. lividus*, Figure 7).

### 2.5. P. lividus PRMT Expression

To study the mRNA expression profile of PRMTs during development, we performed RT-qPCR experiments at different developmental stages.

Results (reported in Table 4) show that, with the exception of PRMT3 and 6, PRMTs are expressed in the unfertilized egg, therefore they are maternally inherited. PRMT1 mRNA resulted hugely transcribed until late blastula stage, while it remained expressed at lower levels at prism stage. Conversely, similar RNA expression levels were detected for PRMT2, 4 and 5, showing a basal expression during embryogenesis. Differently, PRMT7 and 3 are developmentally regulated, rising progressively until early and late blastula stage respectively.

### 2.6. Predicted 3D Structural Model of P. lividus PRMT1

Recently, several lines of evidence [22,76,77,78] suggested that α- and β-tubulins undergo monomethylation or asymmetrical dimethylation in ω-N manner at specific arginine residues. To define methylation pattern occurring in *P. lividus* tubulins, sequence and pattern recognition analyses were carried out to map and characterize the amino acid residues putatively methylable and the kind of modification (monomethylarginine, MMA; asymmetric dimethylarginine, ADMA, or symmetric dimethylarginine, SDMA) (Table 5).

Based on the conservation of type-specific methylation consensus, we deduced that also in *P. lividus* tubulins the major modifications can be represented by MMA and ADMA. Such modifications are catalysed by type I PRMTs and particularly by PRMT1 which is known as the predominant type I PRMT in vertebrates [86] and also during *P. lividus* embryo development. Therefore, computational analysis tools were herein used to define the 3D structure of the PRMT1 orthologue in *P. lividus*.

**Figure 8:**
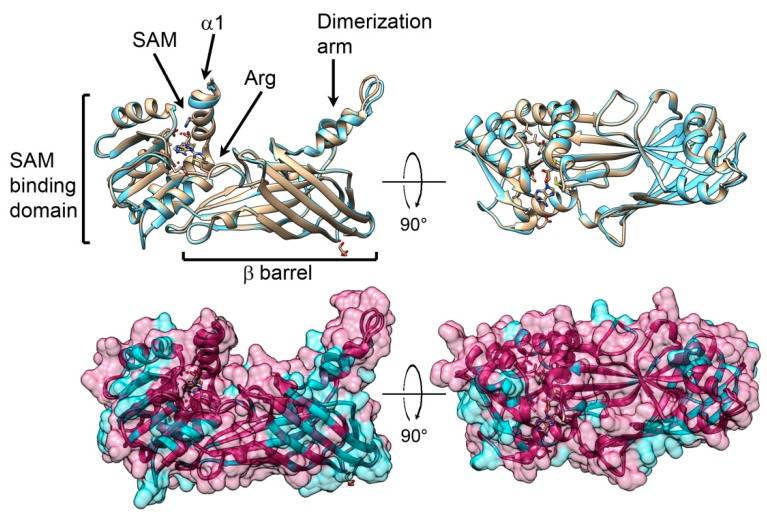
Molecular evolution of sea urchin PRMT1. Top: Superposition of three-dimensional models in ribbon representation of rat PRMT1 (1ORH; showed in pale) and *P. lividus* PRMT1 showed in light blue. Bottom: same superposition in surface (50% transparency) representation with their structures coloured according to the evolutionary conservation of amino acids. The *P. lividus* 3D structure was created via the Phyre 2 software [87] and rendered by using Chimera package [88]. Variable positions are presented in blue; while conserved amino acids are shown in red as defined in the colour-coding bar. In all the structures only the residues after amino acid 40 are shown, since the amino terminus is disordered.

**Figure 9:**
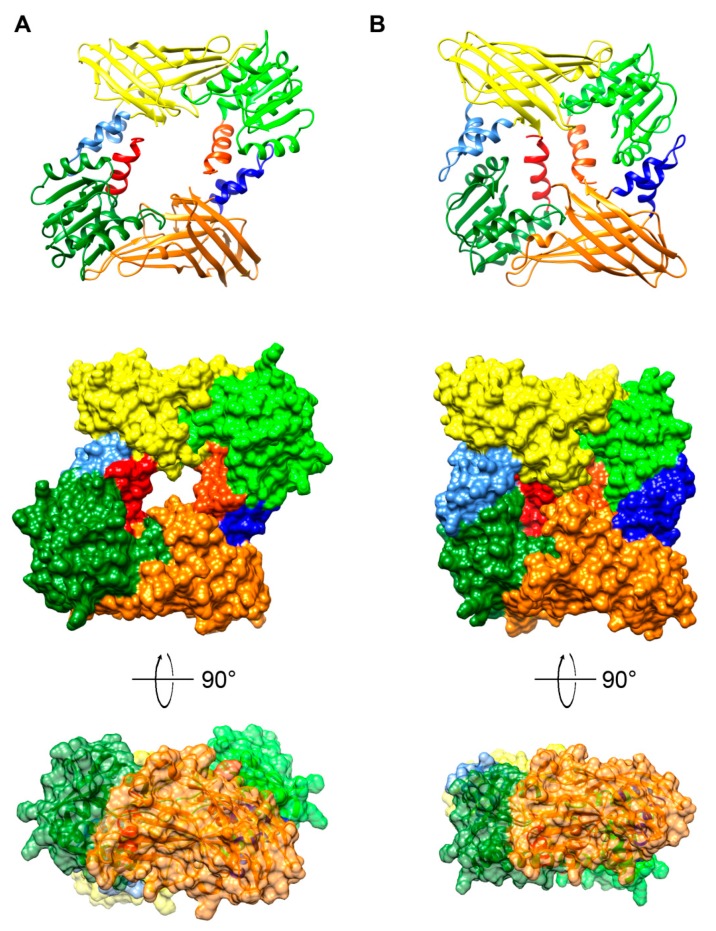
*P. lividus* PRMT1 dimer structure models obtained by ClusPro [89]. The colour coding is red/orange red for the N-terminal domains, green/light green for the SAM binding domains, orange/yellow for the β barrel structures, and blue/light blue for the dimerization arm. (**A**) the ring-like model similar to the model described in Zhang and Cheng [85]; (**B**) the best ranked compact model.

On the basis of the computational analysis, we determined the key features of *P. lividus* PRMT1 (Figure 8). Results gave an extremely high accuracy model, with 89% of residues modelled at >90% confidence and only the 40 N-terminal residues, expected disordered, were modelled by ab initio calculations, therefore they are not shown in structure Figure 8 and Figure 9. The results highlight the typical two-domain structure—a SAM binding domain and a barrel-like domain—with the active site pocket located between the two domains.

*P. lividus* PRMT1 structure was compared to the known rat structure [85]. There are few structural changes among the protein structures, consisting of a slight difference in the orientation of the loop in the dimerization arm and a loop between β14 and β15 (Figure 8).

To recreate the structure of a functional enzyme, a *P. lividus* PRMT1 dimer was computed by molecular docking avoiding use of the unstructured N-terminal region which could impair the reliability of the model (Figure 9).

The resulting models using the balanced coefficients were compared to the available dimer structure already reported [85]. Interestingly, even if a model recapitulates the already published dimer structure of rat PRMT1 [85], other significant structures were also retrieved. We report in Figure 9 the best ranked model which appears more compact and with additional contacts between monomers, resulting in a loss of the cavity in the ring-like structure (Figure 9B).

### 2.7. Tubulin Arginine Methylation

**Figure 10:**
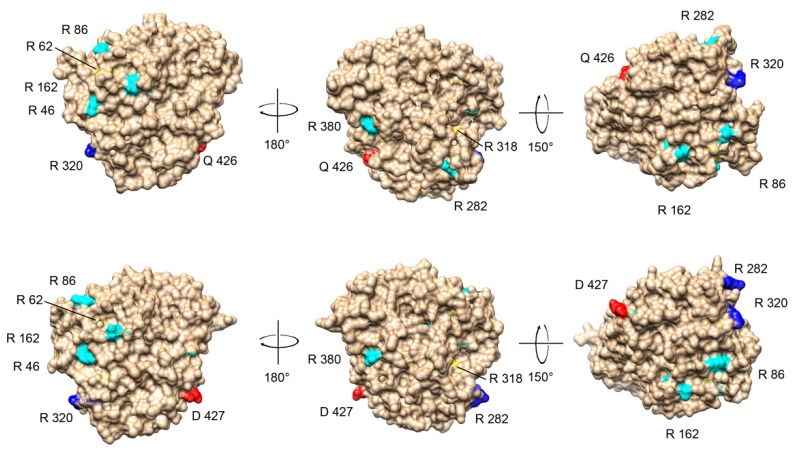
Comparison between arginine methylation sites in human and *P. lividus* β-tubulins. Top, ribbon/surface diagrams of the human neural β-tubulin structure Tubb3 (5JCO). Bottom, ribbon/surface diagrams of the neural β-tubulin structure generated by homology modelling. The 3D structures were created via the Phyre 2 software [87] and rendered by using Chimera package [88]. The arginine residues that can be methylated were coloured according to their accessibility on the surface: buried in yellow, intermediate accessibility in cyan, exposed in blue. The last amino acid shown in the structures (Q426 for the human protein and D427 for the *P. lividus* protein) are coloured in red as reference.

Methylation on R339 of some α-tubulin isotypes and R46, R62, R86, R162, R282, R318, R320 and R380 of some β-tubulin isotypes were recently shown by proteomics study of protein methylation (for details see Table 4) [22,76,77,78]. To provide a comprehensive survey of computed methylable aminoacid residues also in the light of physical constraints, we superpose the *P. lividus* tubulin 3D structures with human orthologues. As expected, structures appear extremely conserved, maintaining secondary and tertiary structure features with RMSD values <1Å. The predicted methylable arginine residues in *P. lividus* structures were characterized in terms of evolutionary maintenance and solvent accessibility to provide functional sites for enzymatic modifications. A representative comparison is shown in Figure 10 where methylable ariginine residues are coloured according to their RSA in *P. lividus* Btub3 and human Tubb3.

According to evolutionary maintenance of selected residues, methylation consensus in primary sequence, organization of secondary structural elements and 3D conformation, arginine residues were similarly computed among the two orthologues, showing a huge conservation in terms of RSA profiles. Therefore, it is reasonable to suppose that MMA and ADMA modifications occurs on sea urchin tubulins in a manner that resembles vertebrate mechanisms. Interestingly, it should be noted that also buried residues which are known to be modified in humans (R62 and R318) are analogously located in sea urchin isotypes, suggesting that changes (including structural modifications after PRMT docking) are likely required for enzyme activity.

## 3. Materials and Methods

### 3.1. Data Mining

The characterized *P. lividus* α- and β-tubulin sequences were used initially to retrieve their corresponding sequences from the publicly available EST database at the National Centre for Biotechnology Information (NCBI). To rescue the expressed isoforms, these sequences were used as queries to perform extensive nucleotide BLAST (BLASTN) and protein-nucleotide BLAST (TBLASTN) searches. The *P. lividus* expressed PRMTs were analogously collected using human PRMT sequences as queries.

### 3.2. Embryo Cultures

Gametes were collected from gonads of the sea urchin *P. lividus* collected in the South-West coast of Sicily, nearby Capo Granitola. Eggs were fertilized at a concentration of 5000/mL and the embryos were grown under gentle rotation at 18 °C in Millipore filtered seawater (MFSW). Developmental stages were monitored by optical microscopy.

### 3.3. RNA Purification, Reverse Transcription and DNA Extraction

Total RNA was extracted from unfertilized eggs and from embryos at 10 (early blastula), 16 (late blastula) and 36 h (prism stage) of development in MFSW using the RNeasy Mini Kit (Qiagen, Hilden, Germany) following the manufacturer’s instructions and performing DNase treatment. RNA concentrations were fluorometrically verified on Qubit 2.0 Fluorometer, while RNA integrity was checked using a 1.5% agarose gel. RNA was stored at −80 °C for future use. An amount of total RNA corresponding to 2 μg was treated with DNAseI, Amplification Grade (Sigma-Aldrich) to remove any residual genomic DNA contamination, and DNAseI was inactivated by adding 50 mM EDTA. First strand cDNA was synthesized from 1 μg DNAseI-treated total RNA samples using SuperScript VILO cDNA Synthesis Kit (Invitrogen), following the manufacturer’s instructions. The cDNA mixture was diluted 1:20 prior to use in Real Time qPCR experiments.

Genomic DNA was extracted from sperm cells from a single animal using the GenElute Blood Genomic DNA Kit (Sigma-Aldrich, St. Louis, MO, USA) and further genomic DNA purification steps were performed according to the manufacturer’s instructions. DNA concentrations and quality were verified by spectrophotometry (OD at 260 nm), whereas the integrity was checked using a 0.8% agarose gel. The DNA was stored at +4 °C for future use.

### 3.4. cDNA Identification

Based on the partial *P. lividus* PRMT, α- and β-tubulin sequences, the 5′- and 3′-end were obtained by PCR-RACE using the 5′/3′ RACE Kit, 2nd Generation application kit (Roche) and the primers (Table 6) as described in the user manual.

The full-length cDNA, consisting of sequences from original ESTs and additional elements from RACE products, were cloned into the pGEM-T Easy vector (Promega, USA) and transformed into XL1- Blue *Escherichia coli* cells (Stratagene, San Diego, CA, USA). Plasmid DNAs were purified on Illustra™ plasmidPrep Mini SpinKit (GE Healthcare Life Sciences, Chicago, IL, USA) and sequenced using T7 and SP6 primers.

### 3.5. RS-PCR and α- and β-Tubulin Gene Organization

Based on the α- and β-tubulin 5′ and 3′ UTR sequences, the primer sets reported in Table 7 were used to isolate the genomic DNA sequences and define the gene structures. Tuba3 gene amplicons were obtained amplifying two fragments (Up and Down).

Moreover, the Restriction-site PCR [90] was carried out to isolate genomic elements adjacent to previously cloned sequences (primer sets are listed in Table 8).

Amplified genomic fragments were cloned in pGEM-T Easy plasmid vector and tubulin clones were fully sequenced by primer walking. Sequences were assembled with Codon Code aligner and were annotated using ab initio or *with similarity* gene prediction programs (i.e., FGENESH using *S. purpuratus* specific gene-finding parameters [91]) and then manually curated.

### 3.6. RT-qPCR

Real-time PCRs were performed in 96 well plates in a 20 μL mixture containing 1 μL of a 1:20 dilution of the cDNA preparations, in the BIO-RAD CFX96 system using the following PCR parameters: 95 °C for 5 min, followed by 40 cycles of 95 °C for 10 s, 60 °C for 30 s. The sequences of the specific primer pairs used for qPCR are shown in Table 9. Samples were run in triplicate, with positive controls (100 pg plasmid clone DNA) and negative controls (no cDNA). The absence of nonspecific products was confirmed by both the analysis of the melt curves and electrophoresis in 2 % agarose gels. To obtain sample quantification, the E^−ΔΔCt^ method was used, and the relative changes in gene expression were analysed as described in the CFX Manager software manual. 18S rRNA was used as internal reference, in order to compensate for variations in input RNA amounts.

### 3.7. Sequence and Structural Analyses

Functional sites and domains in the predicted amino acid sequences were predicted using the InterProScan software [92].

MSA were constructed using T-Coffee (Tree-based Consistency Objective Function For alignment Evaluation) [93]. Phylogenetic and molecular evolutionary analyses were conducted using a Neighbour Joining (NJ) method, implemented in MEGA version X [94] in which Poisson correction, pairwise deletion and bootstrapping (1000 replicates) were considered as parameters, to define the diversification of different protein families herein analysed. Moreover, the 3D structures were reconstructed by homology modelling via the Protein Homology/analogY Recognition Engine 2.0 (Phyre 2) software [87] using the intensive modelling model. Candidate structures for homology modelling were selected according to pairwise alignment. At least two different structures were used as a template for each generated structure as reported elsewhere [82,95,96,97] and extremely high accurate homology models were built for all the sets of proteins. Secondary structure assignments and relative solvent accessibility (RSA) were calculated by the DSSP program as implemented in ENDscript [98]. Additionally, the evolutionary variability of amino acids onto the reconstructed structures were rendered using the UCSF Chimera package [88].

The Multimer Mode Docking option of ClusPro tool [89] was used to compute the dimer structure of *P. lividus* PRMT1, with the number of subunits set to 2 and selecting the option “Remove Unstructured Terminal Residues”.

## 4. Conclusions

In the present work we provide a comprehensive analysis of the expressed α- and β-tubulin gene families in the *P. lividus* model system. Tubulins are recognized targets for antiproliferative drugs used as chemotherapeutic agents. To date, several lines of evidence suggest their use for drug assessing and toxicity evaluation. Moreover, effectiveness of MT binding compounds has been shown to rely often on different PTM degree on tubulins, thus providing different or controversial issues. Among PTMs, methylation carried out by PRMTs were recently associated to drug resistance mechanisms. In particular, some α- and β-tubulin mutations that impair taxanes and epothilones binding to tubulin include arginine residues that undergo methylation (β282^Arg→Gln^) [99]. Moreover, PRMTs are known to be expressed with different subcellular locations in different cancer types and on the other hand, it has been reported that methylation of R282 reduces Taxol binding [22]. Therefore, it has been suggested [23] to use PRMT inhibitors in combination with classic chemotherapy drugs to overcame Taxol resistance. In cancer- targeted therapies, cell division could be inhibited impeding arginine methylation of tubulins, providing full access to Taxol. Alternatively, drug development may also require the production of Taxol derivatives that bind also to methylated tubulin.

Herein, we show that the tubulin and PRMT system in *P. lividus* extremely resembles the mammal counterpart, in terms of sequences, phylogeny and structures. Additionally, chemical-physical constraints on modifiable arginine residues resulted conserved among the systems.

*P. lividus* embryo is a widely recognized model for several research programs spanning from cell proliferation assays [100] and differentiation patterns to toxicological studies [56,57,61,101,102,103,104,105,106,107,108], and also in poor-resource settings. Moreover, the sea urchin embryo has been also intensively used in order to select antimitotic compounds (and glean insights into the biological mechanism) from libraries of 1,3,4-oxadiazole derivatives [43], combretastatin derivatives [44,45] and analogues [109,110] and also 3-amino-thieno[2,3-b]pyridines [111].

Herein, we provide additional elements to extend the reliability of the sea urchin embryo also to MT targeting drugs that are affected by arginine methylation. Transfer of nucleic acid, proteins and drugs performed by microinjection in egg or in one of the blastomere of the early stage embryos are established procedures [112,113] that can allow the modulation of drug actions and responses in order to comprehend the activity of targeted elements and to choose a specific compound. Therefore, experimental set up based on modulation of selected PRMTs and tubulins expression level (by means of miRNA or mRNA transfer experiments) could result in the identification of arginine methylation non-sensitive MT targeting drugs. Moreover, local injection of drugs and their derivatives in different blastomeres allow the observation of local effects on the mitotic apparatus [42].

Additionally, in times of the complex identification of the proper animal system due to ethical issues, *P. lividus* embryos solve many problems also in accordance with international and local laws. This offers the opportunity to test novel anti-proliferative drugs in the nearest non-chordate deuterostome relative, thus providing an animal system supporting cancer research programs including those “from the bench to the bedside”.

## Figures and Tables

**Table 1:** The *P. lividus* α- and β-tubulins.

	mRNA Length (Kb)	ORF Length (nt)	Protein Length (aa)	Molecular Weight ^a^	pI ^b^
Atub8	1.9	1359	452	50,189.55	4.90
Tuba1f	1.7	1359	452	50,186.66	4.90
Tuba1e	1.7	1356	451	50,087.57	4.93
Tuba1g	1.8	1359	452	50,206.67	4.90
Tuba1h	2.0	1359	452	50,208.64	4.90
Tuba1a	1.5	1359	452	50,192.64	4.90
Atub3	1.5	1356	451	50,212.59	4.91
Tuba1d	1.6	1359	452	50,243.73	4.88
Tuba3	2.4	1362	453	50,407.98	4.83
Tuba1b_1	1.7	1359	452	50,353.76	4.97
Btub2	1.9	1344	447	50,051.16	4.73
Tubb2a	1.8	1344	447	50,051.16	4.73
Btub3	1.7	1344	447	50,033.13	4.73
Btub5	1.9	1341	446	49,990.17	4.79
Btub6	1.8	1341	446	49,963.08	4.74
Btub9	2.0	1341	446	50,000.08	4.72
Btub4	1.9	1338	445	49,837.91	4.76

^a^ Molecular weight of the deduced polypeptide in Dalton. ^b^ Isoelectric point of the deduced protein.

**Table 2:** Tubulin genes: core promoter elements and expression analysis during development.

	Core Promoter Elements			Expression			
Gene Name	BRE	TATA	INR	Egg	Early Blastula	Late Blastula	Prism
Atub8			+	+	++	+	+
Tuba1f			+	-	-	+	-
Tuba1e	+		+	-	-	-	++
Tuba1g			+	+	+++	++	++
Tuba1h			+	+	++	+	+
Tuba1a		+	+	-	+	++	++
Atub3		+	+	-	-	-	+
Tuba1d		+	+	-	-	-	+
Tuba3		+	+	-	-	++	+
Tuba1b_1		+	+	+	-	-	++
Btub2	+		+	-	++	-	+++
Tubb2a			+	-	+++	++	++
Btub3		+		-	++	-	+++
Btub5			+	-	+	++	++
Btub6		+	+	-	-	-	++
Btub9	+		+	+++	++	+	+
Btub4		+	+	-	-	-	+

**Table 3:** The *P. lividus* PRMTs.

	mRNA Length (Kb)	ORF Length (nt)	Protein Length (aa)	Molecular Weight	pI
PRMT1	1.6	1077	358	41,123.81	5.33
PRMT2	3.0	1353	450	50,762.22	5.06
PRMT3	2.2	1647	548	62,149.50	4.60
PRMT4	3.4	1185	394	68,586.26	6.77
PRMT5	2.5	1881	626	71,161.21	6.16
PRMT6	2.4	1854	617	44,301.14	5.04
PRMT7	3.1	2061	686	77,028.46	5.55

**Table 4:** PRMT genes and their expression during development.

	Expression			
Gene Name	Egg	Early Blastula	Late Blastula	Prism
PRMT1	+++	++++	++++	++
PRMT2	+	+	+	-
PRMT3	-	+	++	+
PRMT4	+	+	+	-
PRMT5	+	+	+	+
PRMT6	-	+	+	-
PRMT7	+	++	-	-

**Table 5:** Arginine methylation sites identified in tubulins of other organisms, their sequence environment (−6/+6 peptide) and corresponding sequences in *P. lividus*.

Experimentally Defined Methylated Residues							Predicted Methylable *P. lividus* Residues			
Site ^1^	Type ^2^	Tissue	Reference ^3^	Protein Name	Accession #	−6/+6 Peptide ^4^	*P. lividus* Proteins	−6/+6 Peptide ^4^	*P. lividus* Protein	−6/+6 Peptide ^4^
α339	MMA	HCT116 cells	[77]	TUBA1B	NP_006073	ATIKTKrSIQFVD	ALL ^6^	ATIKTKrTIQFVD	Atub8	ATIKTKrSIQFVD
α339	MMA	HCT116 cells	[77]	TUBA4A	NP_005991	AAIKTKrSIQFVD			Atub3	ANIKTKrTIQFVD
α338	MMA	2,9 F ^5^	[77]	TUBA3C	XP_486246	ATIKTKrTIQFVD				
α339	MMA	2,9 F	[77]	TUBA1B	P05213	ATIKTKrSIQFVD				
α339	MMA	2,9 F	[77]	TUBA4A	P68368	AAIKTKrSIQFVD				
β46	MMA	2,0 F	[77]	TUBB2A	Q7TMM9	SDLQLErINVYYN	ALL	SDLQLErINVYYN		
β46	MMA	2,0 F	[77]	TUBB2B	Q9CWF2	SDLQLErINVYYN				
β62		Neuro2a cells	[22]	TUBB3	Q13509	SHKYVPrAILVDL	ALL ^6^	GGKYVPrAVLVDL	Btub6	GGKYVPrAALVDL
β86	MMA	2,7 F	[77]	TUBB2A	Q7TMM9	PFGQIFrPDNFVF	ALL ^6^	PFGQIFrPDNFVF	Btub4	PFGQIYrPDNFVF
β86	MMA	2,7 F	[77]	TUBB2B	Q9CWF2	PFGQIFrPDNFVF			Btub6	PFGQIFrPDNFIF
β86	MMA	2,7 F	[77]	TUBB2C	P68372	PFGQIFrPDNFVF				
β86	MMA	2,7 F	[77]	TUBB4	Q9D6F9	PFGQIFrPDNFVF				
β86	MMA	2,7 F	[77]	TUBB	P99024	PFGQIFrPDNFVF				
β162	ADMA	mouse brain	[77]	TUBB	NP_035785	REEYPDrIMNTFS	ALL ^6^	REEYPDrIMNTFS	Btub4	REEYPDrVMNTFS
β282		Neuro2a cells	[76]	TUBB3	Q13509	RGSQQYrALTVPE	ALL	RGSQQYrALTVPE		
β318	MMA	HCT116 cells	[77]	TUBB2C	NP_006079	TVAAVFrGRMSMK	ALL ^6^	TVAAIFrGRMSMK	Btub5	TVAAMFrGRMSMK
β318	ADMA	mouse brain	[77]	TUBB	NP_035785	TVAAVFrGRMSMK				
β318	DIMETH	*S. cerevisiae*	[76]	YFL037W	P02557	TVAAFFrGKVSVK				
β320	ADMA	mouse brain	[77]	TUBB	NP_035785	AAVFRGrMSMKEV	ALL ^6^	AAIFRGrMSMKEV	Btub5	AAMFRGrMSMKEV
β380	MMA	HCT116 cells	[77]	TUBB8	NP_817124	IQELFKrVSEQFT	ALL	IQELFKrISEQFT		
α79	MMA	root tissue ^7^	[78]	α tubulin	XP_010044940	TV(I/V)DEVRSGTYRQ				
β162	MMA	root tissue ^7^	[78]	β tubulin 5	NP_001289666	REEYPDRMMLTFS				

^1^ Site of Methylation. ^2^ Type of methylation. ^3^ Reference. ^4^ Methylable site environment; differences are in red. Lower case r is the methylated residue. ^5^ F: Fold Change_ Brain/Embryo - mouse. ^6^ All *P. lividus* proteins except for proteins indicated in the next columns. ^7^ Plant tubulins [*Eucalyptus grandis*].

**Table 6:** Oligonucleotides used as primers for the PCR-RACE.

Target	Forward Primer - 3′RACE	Reverse Primer- 5′RACE	Ta ^1^
Atub8	TATACCAACTTGAACCGTC	AGATGAGAAATCCTTGGAGA	48
Tuba1f	ATCTATGATATATGCCGTCG	GACATGGACTGAGATACATT	47
Tuba1e	ATTACGGAAAGAAGTCCAAG	ACTGAAGAAGGTGTTAAAGG	48
Atub3	CTGTTGTCGAGCCATATAA	GCATAGTTATTAGCAGCATC	47
Tuba1d	CTGTAGTTGAGCCCTATAAC	ACTGAGATACATTCCCTCAT	48
Tuba3	GGAGAAGGACTATGAAGAAG	AAGGATTGAGTTGTATGGTT	47
Tuba1b_1	GAATCCATTTCCCTCTTGTA	TATCCATGACTCTGTCAATG	47
Btub6	GATATCTGTTTCCGTACCTT	CTGTAGCCTCATTGTAGTAG	47
Btub9	CCTGAATCATCTCGTATCAG	CACGCATAATGATACAGATG	47
Btub4	AGCTCTGTACGATATTTGTT	CAGCTTGTAGATGAACAATC	47
PRMT2	ATAGAAGGTACAACCCTACC	ATTGCTTCCATCTCCATTAC	48
PRMT3	CAAGGATAAGGTGGTGTTAG	TGGAGAGGAGAAGATTTCAT	48
PRMT4	TGAGCAGGTTGATATCATTG	CTTTGTGTTTAGTGTGTGTG	48
PRMT5	CAAATGTGCTATCTTCTCCA	TTTCCTTCTACGAACTCTCT	48
PRMT6	AGTCCTCATTTACATTCAGC	CCTAGGAGGCTTAGTAGAAG	48
PRMT7	CTACATATCCACATGCTCAC	AGGGGATGTTCACCATATTA	48

^1^ Ta: annealing temperature. PRMT1 coding sequence was already complete.

**Table 7:** Oligonucleotides used as primers for the genomic DNA amplifications.

Name	Forward Primer	Reverse Primer	Ta ^1^
Atub8	CGGCACATCGGACACTGTGA	AGCCGTGGGCGTTGATTGAT	58
Tuba1f	TGTAGCGAGCGACTCAAGCG	TGAGCAAAAGGAGGCTACGGC	58
Tuba1e	ACCCATCCATCACTTGGCACG	TCCCCACATTTTGGCGAATGA	55
Atub3	CGACGTCTGCTAGCTCACCTTA	TGGTTTATTGATCAGCTCTCATGGC	56
Tuba1d	GACGCTTCGCAGCTCTGTCT	TGCATCGAAGGAAGGGGGAT	56
Tuba3_UP	GTCTCGCCGATTTCGCCACT	TGTGCTTGCCAGCTCCAGTC	58
Tuba3_DW	GCGAACCGGTACGTATCGTCA	TTCATCGTAGAATCTTGGAACGCC	56
Tuba1b_1	CGCATCGAACACGGCTCTGA	GGTATCGTGGCCGTGCGAT	58
Btub6	CCTTCTAAGCGAATTGCAAGGTG	TCTGCTTGTACATGCTGCCAGA	55
Btub9	GACGGTTGCACAGCATGCAC	GTCCGGACGCAAGAGTGGTC	58
Btub4	GGTCTGCTTGTGTGTCCCCG	TAACGAGTCCGACCAGGGGG	58

^1^ Ta: annealing temperature. UP: up fragment amplification, DW: down fragment amplification.

**Table 8:** Oligonucleotides used as primers for the RS-PCRs.

Target	Forward Primer - 3′RSO	Reverse Primer- 5′RSO	Ta ^1^
Atub8	TTATAAGGCAACCTCCAACC	GTTTCACAGATTGCTCACAG	50
Tuba1f	ACAACGAACTAAAGGCTCAT	GTATGATGTGCCGTAAGCTA	50
Tuba1e	GCCAAAATGTGGGGATTAAG	TAGACGGATTTGAGGCAAAA	50
Atub3	TAGAATTCAGCCATGAGAGC	TTTGAGGAGCTTTGACAACT	50
Tuba1d	AATCGTCAACAGTTTGCTTC	TATTGAAGAGACAGAGCTGC	50
Tuba3	TTCTGACTTCTTAGTGCCTT	AGTAGAAGTGGCGAAATCGG	50
Tuba1b_1	AGAGGAAGATGACGATTGTG	ATAGGTTAGGCTTCAACAGC	50
Btub6	CACTTGAAAGGAACATCTGC	AATAATGCACAGGAGAGGTG	50
Btub9	TTCCAAAACATTTGCCTGTC	AATGGCGGAAAAATTGTGTT	50
Btub4	CGACCTGTGGATACATCATT	AGATGTAAAGAAACGGGGAC	50
RSO-Bam	TAATACGACTCACTATAGGGAGANNNNNNNNNNGGATCC		
RSO-Eco	TAATACGACTCACTATAGGGAGANNNNNNNNNNGAATTC		
RSO-Nde	TAATACGACTCACTATAGGGAGANNNNNNNNNNCATATG		
RSO-Xba	TAATACGACTCACTATAGGGAGANNNNNNNNNNTCTAGA		
RSO-Sau	TAATACGACTCACTATAGGGAGANNNNNNNNNNGATC		
RSO-Taq	TAATACGACTCACTATAGGGAGANNNNNNNNNNTCGA		
RSO_T7	TAATACGACTCACTATAGGGAGA		50

^1^ Ta: annealing temperature.

**Table 9:** Oligonucleotides used as primers for the qPCR.

Name	Forward Primer	Reverse Primer
Atub8	TGAGCAATCTGTGAAACCTCCTC	TACAACTCCCAGCAGGCATTACC
Tuba1f	CTTACGGCACATCATACGTTGC	CGACATGGACTGAGATACATTCCC
Tuba1e	CTGAGCATTTTGCCTCAAATCC	TCCGACGTGGATAGAGATACATTC
Atub3	CAGTGCAGTTGTCAAAGCTCCTC	GGCTGGATACCATGCTCAAGAC
Tuba1d	AAAGCTCACTTCAAGACGCTTCG	CGACATGGACTGAGATACATTCCC
Tuba3	GCCGATTTCGCCACTTCTACTTAG	AGGCATTTCCCATCTGGACAC
Tuba1b_1	TCTTCGTTGCTGTTGAAGCC	TCAAGGCAATAGAGTTCCCAGC
Btub6	CCTAAGCAAATTGCAGGGTGTAAC	GCCTGTAAATGTACAATCTCACGC
Btub9	CCACGAACACAATTTTTCCG	CCAGCTTGTAAGTGAACAATCTCAC
Btub4	TTTCCCAAAGAGTCGTGGTG	CCAGCTTGTAGATGAACAATCTCAC
PRMT1	GGGAGGACAGGGGGACGGAC	CAGCCCCAGCTTTGGCAGCA
PRMT2	GCCTGGCGGAAATGGGGGAG	TTCCCTTGCGTGCCCACCAC
PRMT3	CTGCTCACCATGGGCGCTGC	AGCCTTCCAATCGGTTTGCGTGT
PRMT4	ACAGCAAGGCAGGGTGGTGC	GCATACATGCCCCCAGCCCC
PRMT5	GTCCGCAGCCGGAGCGTATG	GGGCATCGAGGGCACCATCA
PRMT6	GGCAGAGCCAGAGCCTGTTGG	GCCGCAATCTCTTCCGCACCT
PRMT7	TCGCTGGCGCCTGGAGTACA	CCTGGGCCTTGAGAATGCAGGG
18S rRNA	GAATGTCTGCCCTATCAACTTTCG	TTGGATGTGGTAGCCGTTTCTC

## Supplementary Materials

Supplementary materials can be found at https://www.mdpi.com/1422-0067/20/9/2136/s1.

## Abbreviations

ADMA	asymmetric dimethylarginine
MAP	microtubule-associated protein
MMA	monomethylarginine
MSA	multiple sequence alignment
ORF	open reading frame
PRMT	protein arginine methyl transferase
PTM	post-translational modification
SDMA	symmetric dimethylarginine
TSS	transcription start site
UTR	untranslated region
