# Supplementary figures and images for: A Survey on Tubulin and Arginine Methyltransferase Families Sheds Light on P. lividus Embryo as Model System for Antiproliferative Drug Development
Source: Int J Mol Sci. 2019 Apr 30;20(9):2136. doi: 10.3390/ijms20092136 (PMC6539552; doi:10.3390/ijms20092136)

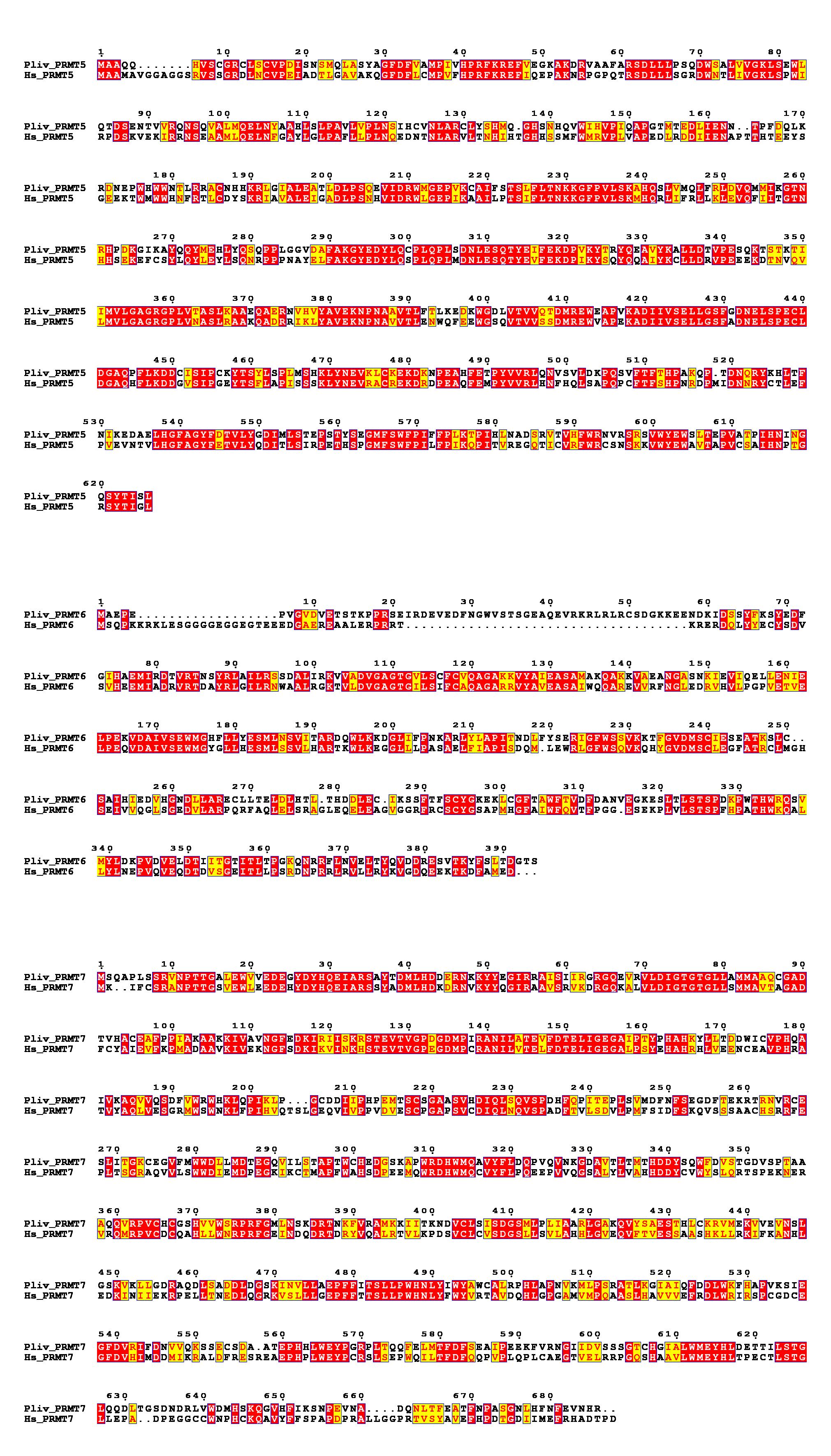

Supplement: Supplementary file 1 [file ijms-20-02136-s001.zip › Figure S2.tif]

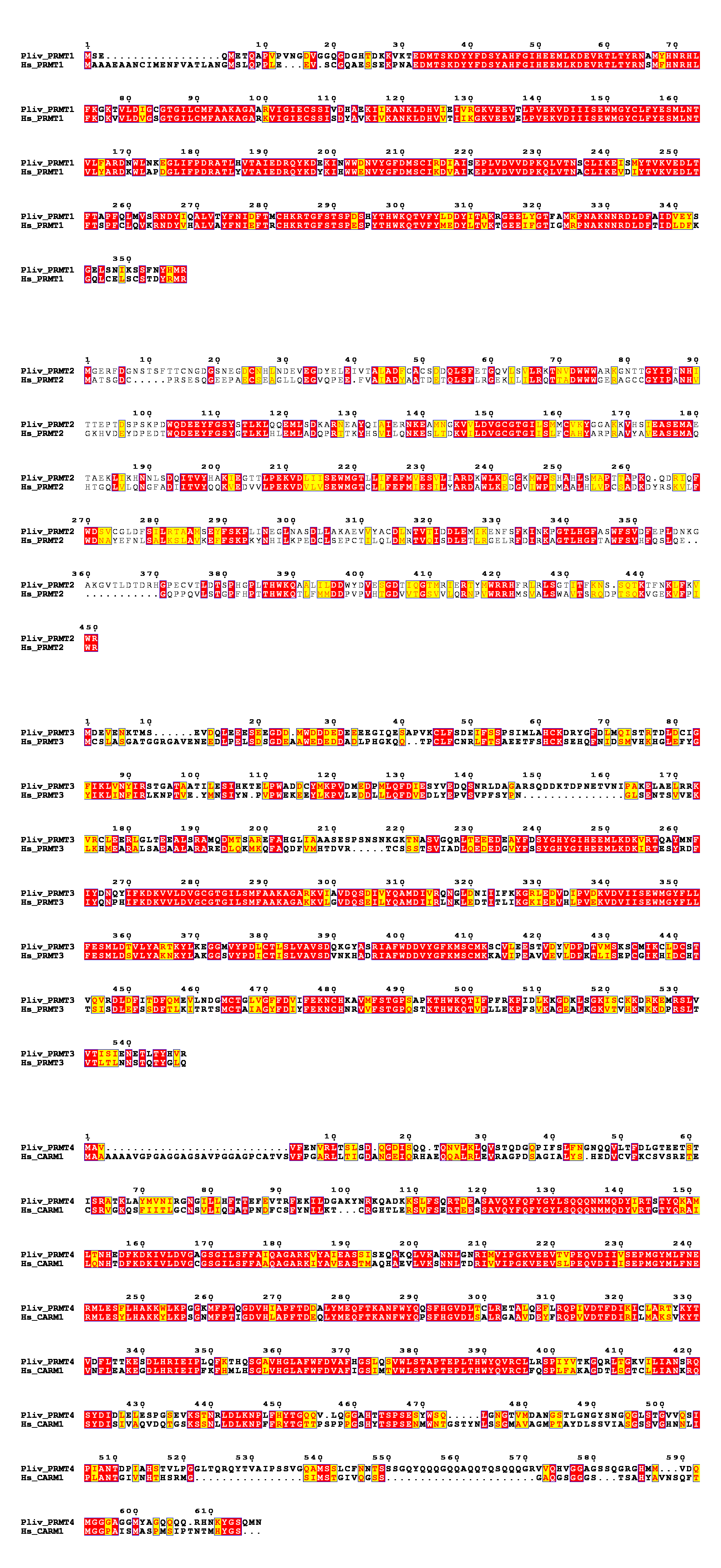

Supplement: Supplementary file 1 [file ijms-20-02136-s001.zip › Figure S1.tif]
